# How collaborative are quality improvement collaboratives: a qualitative study in stroke care

**DOI:** 10.1186/1748-5908-9-32

**Published:** 2014-03-11

**Authors:** Pam Carter, Piotr Ozieranski, Sarah McNicol, Maxine Power, Mary Dixon-Woods

**Affiliations:** 1 Social Science Applied to Healthcare Improvement Research (SAPPHIRE) Group & East Midlands, Research Design Service, Department of Health Sciences, University of Leicester, 28 Princess Road West, Leicester LE1 6TP, UK; 2 Department of Social and Policy Sciences, University of Bath, Bath BA2 7AY, UK; 3 Education and Social Research Institute, Manchester Metropolitan University, 799 Wilmslow Road, Didsbury, Manchester M20 2RR, UK; 4 Salford Royal NHS Foundation Trust, Stott Lane, Salford M6 8HD, UK; 5 Department of Health Sciences, University of Leicester, 22-28 Princess Road West, Leicester LE1 6TP, UK

**Keywords:** Quality improvement, Collaborative, Stroke, Qualitative, Implementation

## Abstract

**Background:**

Quality improvement collaboratives (QICs) continue to be widely used, yet evidence for their effectiveness is equivocal. We sought to explain what happened in Stroke 90:10, a QIC designed to improve stroke care in 24 hospitals in the North West of England. Our study drew in part on the literature on collective action and inter-organizational collaboration. This literature has been relatively neglected in evaluations of QICs, even though they are founded on principles of co-operation and sharing.

**Methods:**

We interviewed 32 professionals in hospitals that participated in Stroke 90:10, conducted a focus group with the QIC faculty team, and reviewed purposively sampled documents including reports and newsletters. Analysis was based on a modified form of Framework Analysis, combining sensitizing constructs derived from the literature and new, empirically derived thematic categories.

**Results:**

Improvements in stroke care were attributed to QIC participation by many professionals. They described how the QIC fostered a sense of community and increased attention to stroke care within their organizations. However, participants’ experiences of the QIC varied. Starting positions were different; some organizations were achieving higher levels of performance than others before the QIC began, and some had more pre-existing experience of quality improvement methods. Some participants had more to learn, others more to teach. Some evidence of free-riding was found. Benchmarking improvement was variously experienced as friendly rivalry or as time-consuming and stressful. Participants’ competitive desire to demonstrate success sometimes conflicted with collaborative aims; some experienced competing organizational pressures or saw the QIC as duplication of effort. Experiences of inter-organizational collaboration were influenced by variations in intra-organizational support.

**Conclusions:**

Collaboration is not the only mode of behavior likely to occur within a QIC. Our study revealed a mixed picture of collaboration, free-riding and competition. QICs should learn from work on the challenges of collective action; set realistic goals; account for context; ensure sufficient time and resources are made available; and carefully manage the collaborative to mitigate the risks of collaborative inertia and unhelpful competitive or anti-cooperative behaviors. Individual organizations should assess the costs and benefits of collaboration as a means of attaining quality improvement.

## Introduction

Quality improvement collaboratives (QICs) seek to address problems in quality and safety of healthcare. In their most characteristic form, they involve teams from multiple organizations coming together to work in a structured way over a limited period to address a specific area of quality of service, often a gap between best practice and actual practice [1,2]. Participants typically take part in a series of meetings facilitated by an expert ‘faculty’ to learn about best practice and share knowledge, experience, and support; they apply interventions to improve quality in their local organizations using specific methods; and they collect and contribute data that enables progress within and between organizations to be tracked. Despite their popularity, evidence for the effectiveness of QICs remains equivocal [3] amid ongoing methodological debates about how they can best be evaluated [4-6]. Though examples of collaboratives that have demonstrated success have been reported [1,7], a systematic review suggests their impact is typically variable and often limited, with only modest evidence of effectiveness [8]. Some work has suggested that only 30% of organizations involved in collaboratives may achieve ‘significant improvements,’ another 30% may drop out, and the remainder may make relatively little progress [1].

The need to identify and characterize what might influence QICs’ achievement of their intended goals is pressing. A recent systematic review identified some of the determinants of success in QICs [3], but more attention is also needed to the question of what interferes with their success. We suggest that a rich social science literature on the challenges of collective action and inter-organizational working may help to explain many of the reasons for the apparent difficulties that QICs experience in realizing their potential, yet it has been under-used to date in the evaluation of QICs. This literature is large and wide-ranging, drawing on contributions from multiple fields, using different terms and concepts according to disciplinary affiliation and applied to diverse substantive areas. It is beyond the scope of this article to provide a full summary; instead, we will highlight an important strand of the literature concerned with issues relevant to inter-organizational collaboration.

Work in this area clearly identifies possible benefits of organizational forms that, like QICs, seek to use collaboration and participatory forms of decision-making and action [9]. Such approaches may not only enable better informed decisions, but also foster collective learning and more sustainable outcomes, unite and motivate those with a commitment to solving problems, increase the chances of detecting the potential for innovation, and improve people’s willingness to accept change [10-15]. Approaches based on principles of collaboration may afford opportunities for practice-based learning and the sharing of ‘know-how,’ as well as ‘know-what’ [7,16,17], and consequently help dissolve inter-professional boundaries that interfere with the spread of knowledge and innovation [17]. Such approaches may, for example, support peer monitoring to improve performance [13] and may be more likely to achieve sustainable impacts through generating a sense of local ownership and commitment [18,19]. Creating links between organizations may stimulate spread of innovation, since organizations with more direct connections to the wider environment are more likely to adopt and institutionalize new models and practices [19]. Overall, structures that enable co-operation and sharing might be said to confer what Huxham terms ‘collaborative advantage’ [20].

Despite the apparently rich potential of collaboration, barriers to co-operation, innovation diffusion, and knowledge-sharing have proved difficult to avoid [21-26]. Many of these challenges arise because human endeavors that rely on large-scale co-operation and coordination are, regardless of setting, susceptible to a range of pathologies. These have been empirically and repeatedly demonstrated in game theory, social psychology, economics, sociology, and related fields [27]. The ‘free-rider’ effect is among the best known of these effects [28]. It arises because some self-interested actors, acting rationally, may substitute their own goals for those of the group, so that collaboration is undermined by the self-interests of individuals as they pursue competitive rather than collaborative advantage [29]. A perhaps less pernicious but nonetheless important problem is that of ‘social loafing,’ where individuals working collectively exert less effort than those working individually [30]. Further potential difficulties arise from either hostility or inertia in the face of the attempted collective effort, an effect known as ‘collaborative inertia’ and defined as slow or non-existent progress [31]. It can arise when the collaborative endeavor is eroded by behaviors such as free-riding and social loafing, but it also occurs because collective action requires time and effort to build coalitions and forge agreement, particularly where local opposition or resistance is found [32]. Efforts that depend on collaboration may start out with much enthusiasm, but gradually develop less helpful features including competition and rivalry between different members of the network, fragmentation or duplication of effort, emergence of disruptive hierarchies, or diminishing commitment and ability to secure resources [33].

QICs are perhaps especially vulnerable to these kinds of problems, as they entail both intra-organizational and inter-organizational collaboration [34]. While such arrangements may generate creative energy [35], the tension between membership of an autonomous organization at the same time as membership of a partnership, network or collaborative is likely to generate paradoxes and transaction costs [31,35-40]. Further, the contexts in which they are introduced, which are often characterized by markets and/or hierarchies [41], may harbor the potential to stifle or overwhelm well-intentioned motives for co-operation. Thus, while collaborative advantage may be a good reason for pursuing collaboration between organizations or groups, it is a complex process fraught with the risk of collaborative inertia [31] and other challenges.

We aimed to draw on concepts from this literature, as well as others derived empirically, to explain processes and outcomes of a QIC that aimed to improve quality of stroke care.

## Methods

We undertook a qualitative study of a QIC known as Stroke 90:10. This QIC, though it reported some improvements in specific outcomes of stroke care across the North-west of England, it also, like many QICs, demonstrated considerable variability in the performance and rate of improvement of the participating organizations [42].

### Intervention

The Stroke 90:10 program comprised a QIC that aimed to improve care for stroke patients. It sought to help 24 participating hospitals to achieve 90% compliance with nine indicators of high quality stroke care by 2010. The nine evidence-based indicators were organized into two ‘bundles’ [43]: Early Hours and Rehabilitation (Table 1).

**Table 1:** Stroke 90:10 evidence-based indicators

**Early hours bundle 1**	
1	Brain imaging within 24 hours of admission to hospital (CT scan) to confirm stroke type (ischaemic or haemorrhagic) and determine management.
2	Delivery of aspirin or alternative antiplatelet (for patients where an antiplatelet is clinically indicated) within 24 hours of admission to moderate stroke complications and improve outcomes. (For shorthand, we refer to this as ‘aspirin’).
3	Swallow screen within 24 hours of admission, to prevent unnecessary withdrawal of nutrition, support timely administration or modification of aspirin/antiplatelet delivery and highlight patients who need on-going management of swallow safety.
4	Weight assessment on admission.
**Rehabilitation bundle 2**	
1	Physiotherapy assessment within 72 hours of admission to improve early mobilisation, and increased likelihood of targeted goal setting.
2	Occupational therapy assessment within four days of admission to support activities of daily living, memory, perception and cognition.
3	Mood assessment (during the in-patient stay) to screen for altered mood and other factors, given that post-stroke depression is known to affect the likelihood of long-term recovery.
4	Documented evidence of MDT goals set for rehabilitation as a marker of patient involvement in care and multidisciplinary team working.
5	50% of the patient’s hospital stay on a stroke unit, defined using the National Audit criteria, given evidence that stroke units reduce mortality and improve patient outcomes.

Stroke 90:10 was based on the Breakthrough Series (BTS) collaborative model developed by the Institute for Health Improvement, which has a philosophy of ‘All Teach, All Learn’ [44]. It adopted the distinctive BTS structure of a faculty of quality improvement experts, three learning sessions (collaborative meetings), and action periods in between learning sessions where participants implemented Plan-Do-Study Act cycles to undertake small tests of change locally and then scale up improvements.

A package of support was available to participating hospitals from the Stroke 90:10 faculty, including executive mentoring visits, direct access to the project director and an improvement advisor, and weekly online sharing and learning sessions via a web based portal (extranet). Learning sessions provided instruction in the theory and practice of improvement, offered teams advice and guidance, and shared cumulative results. Chief executive officers of participating hospitals were asked to commit to releasing staff to take part in collaborative activities over the course of the program.

All invited hospitals participated in the collaborative, but they did so in two phases for purposes of detecting the effect of the collaborative on improvement. Once the first phase - which lasted 12 months - was over, all hospitals entered the collaborative. During the first phase, the hospitals that were awaiting entry acted as a control group for those already in the QIC. Two hospitals withdrew from the first phase and one did not submit sufficient data for analysis, leaving nine hospitals. Similarly, sufficient data were available from nine hospitals in the second phase.

Quantitative evaluation of the QIC reported elsewhere [42] showed a modest advantage of the collaborative for both the early hours and the rehabilitation bundles when the nine first phase hospitals were compared with the nine hospitals that formed the control group.

### Study design

Preliminary analysis of the quantitative data from Stroke 90:10 was completed approximately one year after the collaborative ended. This analysis suggested not only that the impact of the QIC had been more modest than hoped for, it also revealed substantial variability in performance between different organizations. It was then decided to commission an additional qualitative study to explore the reasons for these effects.

Research ethics committee approval was obtained for the qualitative study separately from the ethics approval for the QIC. All hospitals that had participated in Stroke 90:10 were invited to take part in a qualitative study to explore their views and experiences of the collaborative. Eleven hospitals agreed to take part and completed the necessary governance approvals to allow the study to take place. We have used pseudonyms to anonymize participating organizations.

### Data collection

Semi-structured interviews were conducted with hospital staff who had been part of the Stroke 90:10 teams at the 11 participating organizations. A focus group was conducted with the QIC faculty team. Participants were asked about their experiences of the QIC, whether (and, if so, how) it had helped them to improve stroke care, and about the features of their organizations that affected their participation and performance in the collaborative. Those who took part in interviews included radiographers, stroke co-ordinators, specialist stroke nurses, occupational therapists, physiotherapists, healthcare assistants, data collection staff, emergency department staff, ward managers, and members of the hospital executive, reflecting the broad range of professionals involved in the QIC. As a secondary source of data, we also accessed project documents including reports and newsletters. These were purposively sampled mostly as a means of identifying background information about the collaborative, but also where appropriate as a way of triangulating emergent themes from the interview data.

### Data analysis

Our analysis was driven by the diagnostic question, ‘how might an understanding of inter and intra organizational processes explain the limited and variable effects of the Stroke 90:10 QIC?’ Data analysis was based on a slightly modified form of the Framework Analysis approach [45]. This is a pragmatic approach that combines both inductive analysis, where themes are generated from the data, with a more structured deductive approach where the data are assessed against pre-selected constructs and themes [45,46].

The interview and focus groups transcripts were fully transcribed and then coded thematically using N-Vivo™ software. Two kinds of themes were used: a first set based on the literature on collective action discussed above, which we treated as sensitizing constructs [47] rather than as rigidly imposed categories, and a second set that we generated by careful reading and re-reading and then creating suitable constructs for capturing the properties of the underlying data. The project documentation was thematically coded using the codes generated for the interview/focus group analysis plus, where appropriate, [48] a small number of new empirically derived codes.

## Results

We report first on benefits of the collaborative perceived by the participants before discussing what they saw as more problematic aspects. The benefits reported were mostly consistent with those seen in other studies of QICs, but we also found evidence of risks of collaborative inertia and tensions between intra and inter-organizational goals, processes, and outcomes that were characteristic of the challenges described in the literature on collective action.

### Collaborative advantage: motivating change

The QIC was valued for how it created a sense of a greater purpose, and enabled people to look out to other organizations as well as in to their own organizations. Many participants welcomed the opportunity to share experiences with others:

…whereas for 90:10 it just felt as though, it felt easier because it was around looking at it for a greater purpose in some ways. Whereas I think sometimes when you're just sitting in your own place, looking at your own staff it, it's good 'cause you're still making improvements, but it's sometimes you can be too inward looking and it can feel as though you're about to disappear up your own work. Whereas when you've got some outlook for it, it makes it in some ways feel more worthwhile that you've done the work and you're sharing it, rather than you're just, as I say just plugging away in your office and it's good, but it feels as though it's good for its own sake. (Consultant, participant 13)

Stroke 90:10 was identified by some participants as supporting the achievement of goals that might otherwise have been too daunting:

‘I don’t think we would have got to where we are today, the whole team, without 90:10. I think we would have been further back. I don’t think we would have been brave enough to think right, we are all going to go for [it]…and I don’t think on our own, that we would have perhaps pushed that far ahead’. (Specialist Nurse, participant 15)

‘And you realize that the problems that you have are the same problems that everyone else has. You are not out there on your own. So you end up working out what you can do about things and feeding off other people really. So those coming together events facilitate that.’ (Stroke Coordinator, participant 32)

The learning sessions, where members of the QIC came together, were seen by some as especially important in creating energy and enthusiasm. These meetings provided the opportunity to build relationships, learn from the experiences of others, and gain access to formal teaching from the faculty team:

‘And actually those meetings were really energizing each time they happened. Because I think in between meetings, you go away and you plod along … And you could get a little bit, start to feel as if you're trudging along a little bit. Whereas then, going back to a bigger meeting, it just felt as though you were a part of something bigger and it did sort of lift you up for the next, the next bit really’. (Consultant, participant 13)

‘Actually that’s what a lot of people were feeding back. That actually the networking and the shared learning was more useful….I found that when it was break or a lunch time you could grab someone and go ‘That’s really interesting ‘cause what you’re saying is what I found when I did that ten months ago…. What do you think of that? Is that the right thing?’ those conversations I found really useful’. (Team Leader, participant 18)

The learning sessions were successful in enabling the teams to share practical experiences related to improving stroke care. Participating hospitals shared documentation (such as descriptions of clinical pathways) and new ideas (such as allowing nursing staff to prescribe aspirin). One team, for example, developed a whiteboard approach to enable visual display of completion of processes of care for each patient, and this idea rapidly diffused throughout the whole QIC when it was shared at one learning session:

The whiteboard idea actually brought us back to having something visual that everyone can see. So that was the main thing I think we took from it and I think from that we now think of using whiteboards for other things as well, so. Like discharge planning board, that kind of thing. So that's the main thing I think we took from it. (Nurse, participant 14)

‘…meeting those other people was really useful and beneficial because you bounce ideas off each other, like everybody stole our whiteboard idea. Something so simple but everybody took it on board. And I think it is good to be able to share that, there is no point in doing something else that someone has done and it works, you might as well steal shamelessly’. (Specialist Nurse, participant 15)

‘[Question:] DID YOU TAKE ANY IDEAS FROM OTHER TEAMS?’

‘Yes, I mean like the pathway, we borrowed it from [Location 1]. You know because we asked, there are so many examples of pathway documents but we wanted something that was in the form of a tick list rather than having to write reams and reams and the [Location 1] one was really good so we shared their document. You know adapted it to fit us, so that was the biggest thing, I think, that we took’. (Stroke Specialist Nurse, participant 15)

Reciprocity, a sense of belonging to a community of practice and a ‘shared repertoire’ [49] was fostered by face-to-face and (for those who had access) electronic communication such as webinars. This sense of belonging was further promoted by the newsletters produced by the faculty team, which regularly featured photographs of the teams, motivational quotations from famous people, patient stories and a ‘team of the month’ feature, as well as the QIC’s logo. Many participants spoke enthusiastically about the collective sense of dynamism that the QIC generated:

‘I was persuaded by the ambition of 90:10. You know, trying to get there sooner than would otherwise have been the case’. (Physiotherapist, participant 28).

‘I'm always keen to do any improvement projects. I think if you've got that kind of structure, some sort of official structure, you've got resources coming in, you've got that profile within your own trust that this is an improvement project in the [region] and we are part of it, the trust is signed up to it, it's an opportunity to be able to do much more than you normally would, just working as a small team’. (Stroke Coordinator, participant 21)

Further collaborative advantage arose through the benefits that participation in Stroke 90:10 conferred on some stroke teams within their own organizations. The QIC required complex multi-disciplinary teams to be created within hospitals between managers, administrators and clinicians, and it also demanded the endorsement of the chief executive officer. Many participants described how the QIC performed an important agenda-setting function, bringing to the fore issues that had previously received little attention and stimulating their organizations to find solutions. Some stroke teams were then able to gain from their organizations the legitimacy and recognition for their activities and priorities that they felt had previously been lacking:

‘And then because we were participating in it [Stroke 90:10], the trust actually took a key part to provide things like physio assessments over the weekends which was not there before’. (Consultant, participant 25)

Well I think it definitely highlighted ourselves within the [hospital]. I think one of the first things we had to do was go and see the Chief Executive about the Stroke 90:10 project and let him know what was involved and how things were going. So that was great and …we didn’t just send the consultant in like you normally would, we made sure a mixture of the team went in that probably hadn’t met him before. …it definitely highlighted our service outside the trust as well’. (Nurse Specialist, participant 1)

### Collaborative advantage: securing improvement through collaborative participation

The QIC stimulated teams to make changes to improve care for their patients. Where stroke teams had complete control over a specific issue, they often resolved it by assigning responsibility to a named individual or by devising a procedure that they could operate within their clinical area:

‘… the mood assessment we decided that our problem with the mood, our mood [assessment] was really quite poor. But what we felt was that what we needed to just do was actually give somebody the responsibility to just do the mood assessment…. And what we did was we literally incorporated the mood assessment into the initial OT [occupational therapy] assessment’. (Physiotherapist, participant 7)

Many improvements, however, entailed more complex re-engineering of organizational processes and systems. For some stroke teams, the new status they gained through Stroke 90:10 was especially important in enabling these to happen. For example, ensuring that patients with suspected stroke were identified quickly on admission to the Accident and Emergency (‘A&E’) department was a goal that Stroke 90:10 helped some teams to realize:

‘We did a lot of education for the A&E staff about the importance of treating stroke as a medical emergency and getting them to triage them a lot quicker, than what they were, so that helped push that process through’. (Specialist Nurse, participant 15)

‘Getting the patients highlighted to the stroke team, getting us into A&E and that was why we developed then the stroke response team. That was something we didn't have that before we did 90:10. It was just [person 1] on her own had the bleep 5 days a week. I don't think she would have gone into A&E. That was something we developed during the 90:10’. (Stroke Nurse, participant 19)

Stroke 90:10 also helped to secure improvement through its audit and feedback functions. QIC participants were expected to collect monthly monitoring data and submit it to the faculty team. This enabled the tracking of compliance with care processes over time and encouraged teams to reflect on their current practice, celebrate success where it was found, and identify areas for improvement when needed:

‘I suppose just to make sure that we feedback to the staff … To show their performance … I mean often it’s actually to give them a pat on the back and tell them how well they’re doing. But also to sort of pick up on anything that we’re missing and make those improvements straight away’. (Physiotherapist, participant 7)

…you could see that you were improving and it sort of gave you the impetus to carry on and also you could see where you were falling down. Like we picked up that we were falling down on weights but when we looked through the notes it was our simple patients we were falling down on, you know, they could stand on a pair of scales that were in for a couple of days and it was like this is madness that we’re failing the bundle on something that would be so simple. So I think the monthly data was worth doing’. (Nurse Specialist, participant 1).

In summary, the Stroke 90:10 QIC demonstrated many of the features of collaborative advantage that the literature identifies as a benefit of collaborative activity. However, the benefits of QIC participation did not accrue uniformly across the collaborative, and there was variable commitment amongst the different organizations. In the next section, we present findings that demonstrate the challenges involved in realizing the aim of collaborative advantage.

### The effort required to collaborate: risks of free-riding, social loafing, and inertia

Cooperation and collaboration, the principles underlying all QICs, were not straightforward to achieve. In part this was because significant effort was required to participate in the QIC: direct and opportunity costs were incurred for all Stroke 90:10 participants. These included time and resources to diagnose current problems, plan solutions and sustain intra-organizational teams. Some of these costs would have arisen in any improvement effort, but others were linked directly to collaborative working across organizations, including time away from the workplace, having to comply with an externally imposed timetable and data collection and submission expectations, and having to learn and use the specific improvement techniques promoted by the QIC. Some participants expressed concern because taking part in the QIC required individuals to contribute time and effort on top of their routine ‘day jobs’:

‘I think all the documentation and everything in between, you know, it was difficult to get on board with all of that. That was […] on top of your day job so to speak’. (Consultant, participant 3)

The extent to which participating teams committed authentically to the collaborative ideal appeared to be variable. Interviews suggested that some teams were more likely than others to attend and participate enthusiastically in meetings and other events and to share ideas and offer support to other teams. These reports could be triangulated with quantitative data in a project report that documented attendance rates and return of monthly monitoring reports. This demonstrated variable participation rates (Table 2). Only two hospitals exceeded 75% participation in learning sessions and webinars. Three hospitals did not participate in any webinars; one hospital did not submit any monthly reports for the duration of the QIC. Even when they did take part, some teams were perceived to invest little energy in collaborative activities, and thus contributed to inertia:

‘And they were there, they were present, bums on seats at the learning sessions, but we just were getting nothing from them’. (Faculty team)

**Table 2:** Overall attendance/participation rates

**Activity**	**Learning session**	**Webinar**	**Monthly report**	**Data collection**
Collaborative average attendance/participation	88%	30%	50%	53%
Highest level of attendance/participation by a hospital	100%	84%	100%	99%
Lowest level of attendance/participation by a hospital	30%	0%	0%	0%

Among QIC participants was a sense that not all teams had given to others to the same extent as they had benefited, and that they were thus ‘free-riding’ or undermining the collective action principle. One team, for example, were described as ‘users,’ ‘keeping secrets’ to themselves and ‘ripping off’ the collaborative by ‘siphoning ideas’ and using them to achieve local improvement results while giving little back. Teams who saw themselves as higher-performing sometimes posed the classic individual versus collective action question of ‘what’s in it for us?’, and were unsure whether the QIC offered them added benefit. Their behaviors might be characterized as a form of social loafing, since they left the task of helping lower-performing hospitals to others:

‘We went in with a mind-set of we’re not quite sure what we’re doing here because we’ve already achieved a lot of what this project is setting out to achieve. So on the 2008 stuff certainly from a therapy point of view we were already hitting 90% [compliance]. So I was going to learn how to improve a service that had already achieved what the professionals were trying to achieve. … And obviously we were already well underway with the trust’s service improvement so we all felt we’d got lots of buy-in and we’d seen lots of improvement already. So we weren’t quite sure where the 90:10 project was trying to take us’. (Manager, participant 17)

### Inequalities and competition as a source of collaborative tension

Participants perceived that some variability in teams’ contribution to the QIC was linked to significant asymmetry in starting positions on entry and the competitive environment in which the QIC was launched. The participating hospitals demonstrated highly variable baseline performance on the nine indicators of quality stroke care that were the focus of the QIC activity, and this contributed to feelings of inequality in status and variability in perceptions of what was to be gained from participation. Some low-performing hospitals wanted to improve and therefore had more opportunity to benefit from participating in the collaborative; others felt that they were already high-performing, and had little to gain. Some higher-performing participants were unconvinced by the imperative to share and help others, expressing concern about duplication of effort and the extent to which the QIC would deliver benefits over changes that they were initiating anyway:

‘Ideas like the whole stroke unit coming together was something that was already in process anyway’. (Physiotherapist, participant 16)

Pressures for centralization of stroke services and associated competitive imperatives further undermined the extent to which collaboration was embraced whole-heartedly by all participants. Some, for example, were bidding competitively to qualify for specialist status at a time when stroke services were being reconfigured regionally to create centers of excellence. Their priorities were not therefore necessarily aligned with the collaborative aim of sharing with others and helping all improve:

‘Because we're a comprehensive stroke center, we had to have certain things in place before all of this, for us to be able… to be in the bidding process. And you had to tick so many boxes to be the center of excellence, if you like. So a lot of these acute issues, we were already managing’. (Nurse, participant 14)

Hospitals also had different starting points in terms of their use of methods for improvement. Though some participants welcomed learning about the improvement approach taught by the faculty team, others were already familiar with and favored their own approach. They were unsure of the value of investing additional effort in learning the new terminology and approaches advocated by the QIC:

‘So it’s… would you go on something that teaches you how to achieve what you’re already achieving?…It’s not that I don’t want to learn…I want it to teach me something that I’m not already doing’. (Team Leader, participant 18)

‘But there wasn’t an **incentive** to do that because we’d already got a service improvement methodology that was working for us…I got what they were talking about but it was sometimes hard to concentrate on it because they were teaching us a way of improving the service when we’d already got a model that we were working with, that we were familiar with, that we were comfortable with. So it was sometimes kind like; well, what’s the point of **investing** in this learning session? Because I’m not gonna be using that anyway’. (Manager, participant 17, emphases added)

References to a need for effort or ‘investment’ in learning and perceived lack of incentives highlight an instrumental transactional approach that involves weighing up the added value of the collaborative. Again, it contrasts with the QIC emphasis on shared goals and shared teaching and learning.

### Intra-organizational support

Problems of difference between teams appeared to become more pronounced as the QIC progressed. Ongoing variability in performance became increasingly visible through the data collected by the QIC as a ‘league table’ effect began to emerge. At the same time as some perceived that their potential for gain from the QIC was limited because they had already improved, and some saw it as a prompt to learning, others felt disappointed and potentially humiliated by having poor performance revealed:

‘We improved on the 9 [indicators] that we'd targeted and we'd really worked hard on. But the trust couldn't see that, all it could see was what was presented to it, because you know, that's what goes out in the public domain, you're in the lower quartile. (Stroke Coordinator, participant 21)

Some of the reasons why some teams found it hard to improve lay in the support they received from their home organizations, which did not always commit fully to the collaborative ideals. Some organizations, as we described earlier, gave priority to QIC activities and supported stroke teams by uniting around shared goals. Others, however, were challenged by multiple competing priorities, resulting in the dispersion of energy and effort and lack of commitment to the QIC. In order to provide endorsement, recognition and practical support for activities relating to the QIC, leaders of stroke team needed to have sufficient authority (‘oomph’) and dedicated time within their role in order to implement and sustain quality improvement. They also needed to prioritize and communicate the importance of the QIC within their organizations, yet the extent to which this occurred in practice varied:

‘We were initially led by a non–clinical member of staff who then left half way through…. Stroke was only a very tiny part of his job. And it wasn’t the main focus of his job … so obviously it wasn’t his main priority. And because he didn’t have the clinical oomph that maybe a stroke coordinator would have. And I think unfortunately as well because there were other things going on in the hospital as well as the stroke thing. And 90:10 didn’t come with particularly- I don’t think the consultant felt it was a particularly important thing’. (Physiotherapist, participant 16)

Improvement activities planned by stroke teams were also vulnerable to being downgraded by senior leaders who were distracted or were committed to other priorities. This created collaborative inertia; stroke teams unable to make improvements because of lack of internal support were then unable to make a meaningful contribution to collaborative activities:

‘The organization was not in the best of places. So the organization was sort of landlocked into a sort of a … battleground for services, for all sorts of things. And they were under the cosh because their activity was high and therefore they weren't achieving some of the basics… So there's a lot of other stuff to distract them from what they should be doing’. (Faculty team)

‘Oh the other thing is, wasn’t [that organization] an aspirant foundation trust? So they hadn't yet achieved foundation trust status and I think that consumes organizations when they haven't. You know the leadership of organizations, that's really all that they want to focus on’ (Faculty team)

The absence of intra-organizational collaboration meant that some staff (in particular those lower in organizational hierarchies) felt disadvantaged at learning sessions and unable to make strategic use of QIC participation to secure change in their local environments:

‘And when they went on the first learning session the three of us that were lower grades felt very out of our depths…And also felt as though we’d been let down I suppose by other members of the team, that they didn’t think it was important enough to have some senior staff involved…Which obviously it came to light when you went and there was a chief exec from somebody’s trust…And we didn’t even have a ward manager’. (Administrator, participant 12)

## Discussion

The Stroke 90:10 quality improvement collaborative did produce many of the benefits traditionally associated with non-hierarchical networks and partnerships [50], including reciprocity, trust, mutuality, and a sense of shared purpose. Many participants attributed added value to the QIC and viewed it as a powerful mechanism for quality improvement. But our findings highlight aspects of QICs that, though well known in the literature on collective action, have been under-recognized in relation to quality improvement. The corpus of academic work on collective action suggests the need to acknowledge both the costs and the benefits of collaboration [38,50] and the ways in which they may be unevenly distributed [51,52]. Risks of free-riding, social loafing, and competitive pressures may be equally or more important in their effects than imperatives to collaborate and share, and may induce collaborative tension and collaborative inertia with consequences for the ability of a QIC to demonstrate improvement across the board. The features of intra and inter-organizational collaborative activity that we have identified in Stroke 90:10 may have powerful influences on the extent to which the ideals of QICs can be realized more generally.

Those considering QICs as an approach to quality improvement may need to consider the risks and benefits of focusing on a single organization versus engaging in collaborative effort with other organizations. It is clear from participants’ accounts that both direct and indirect costs arise; some are linked to improvement work (and thus would be incurred whenever efforts are made to improve) but others are linked specifically to collaborating with other organizations. These costs were often rationally weighed up against the benefits of sharing and participating fully in the collaborative. This transactional behavior is well-recognized in the literature on partnership working [31,35,37,38] but has often remained obscured in writing on QICs. Because of the effort involved and the inherent complexity, Huxham [35] goes so far as to urge organizations not to collaborate unless there is a clear collaborative advantage to be gained. Given that improvements can be achieved without collaboration [53], and that one study in a single organization reported impressive results in improving stroke care over a four year period [54], it is therefore sensible for a full assessment of the benefits and costs of collaborative working to be made at the outset of an improvement effort. Organizations may also wish to consider whether more limited forms of collaboration, perhaps involving reciprocal peer review between pairs of organizations, might best meet their improvement goals [55].

Where inter-organizational collaboration is embraced, our findings point to several features of QICs that are important to anticipate and manage. Though overall participants reported a high level of sharing and commitment to a collaborative ethos, these were not universal features of the QIC. In contrast to the democratic principles implied by many QIC models and by the ‘all teach, all learn, all improve …’ [34] philosophy, some participants were more prepared than others to both teach and learn. The external context was important in this. The Stroke 90:10 QIC operated within a context of a competitive market place for delivery of stroke services, and participants did not enter a level playing field; they had very different starting positions and ability to achieve their aspirations. The local or ‘internal’ context is clearly also highly influential. For any project team to be effective, clarity about membership, leadership, continuity, and the ability to work through differences and deal with conflict is required [56]. Our study shows variability in how intra-organizational teams in Stroke 90:10 were configured, with some more supported than others by senior leaders and some more functional than others. The benchmarking data collection system created a ‘league table’ of apparent winners and losers, which was in tension with the collaborative ethos and the expressed desire to learn from failure. Whether benchmarking is experienced as friendly rivalry or as stressful and humiliating may depend upon the degree of trust between participants but also on how data is reported in the public domain. Where there exists a lack of trust and a lack of incentives (financial and/or symbolic) for those participants who are ahead of the game to enhance the performance of others, trust may not be sustained, and the reciprocal principles underlying the QIC may be eroded. Recent work has found that collaboratives may have the greatest effect on those participating organizations with a low baseline of adherence to best practices [57]. The potentially damaging effects of competition on QIC activity have also been recognized in a recent qualitative case study of collaborative quality improvement in intensive care units (ICUs) [58]. Our study points to the risk that organizations that are high-performing at baseline may withhold effort or fail fully to support others, especially in a the context of a competitive external environment.

### Study limitations

Our qualitative study was conducted in an effort to understand why the Stroke 90:10 collaborative had not produced more striking success. Given that this qualitative study was designed and conducted as the quantitative findings had begun to emerge, interviews were not, as would have been ideal, undertaken concurrently with the collaborative. Issues with recall may therefore have occurred. It was not possible to undertake a formal check on theoretical saturation as the opportunities for theoretical sampling were constrained by availability of participants. We cannot be certain that our findings are generalizable across all participants in Stroke 90:10. Though valuable knowledge can be generated *posthoc*, as our study has shown, future studies of collaboratives would benefit from an evaluation design that integrate qualitative and quantitative methods from the outset.

## Conclusions

Previous work has shown equivocal evidence in favor of generating collaborative advantage though quality improvement collaboratives. Our study, informed by concepts from the literature on the challenges of collective action, demonstrates some of the challenges associated with implementing collaborative processes. Fundamental tensions exist where there are competing agendas between individual organizations, professional associations, management imperatives and collaborative aims [31,35]. Organizations should take into account context, costs, time, and other available resources, and set realistic targets when planning quality improvement. Collaboration is not the only type of behavior that occurs within a QIC; those planning QICs in the future should anticipate other behaviors too.

## Competing interests

Maxine Power was a member of the faculty of the QIC under study.

## Authors’ contributions

MP designed the intervention and was part of the multi-disciplinary team that conducted the RCT to evaluate the intervention. MDW and MP conceived of and designed the qualitative study. SM carried out the interviews, undertook preliminary coding of the data, and produced interim reports. PO also coded and analyzed the data. PC conducted the literature review, developed the analysis and led on drafting of the manuscript, with extensive contributions from MDW. All authors read and approved the final manuscript.

## Access to data

PC, PO, SM, and MDW had full access to all interview data. For ethical reasons it was not possible to share all raw data with MP.
